# Pregnancy-Associated Chest Pain: A Case of Spontaneous Coronary Artery Dissection

**DOI:** 10.1155/2021/4057182

**Published:** 2021-01-15

**Authors:** Vidhushei Yogeswaran, Satvik Ramakrishna, John S. MacGregor, Lucas Zier, Nora Goldschlager

**Affiliations:** ^1^Department of Medicine, University of California, San Francisco, CA, USA; ^2^Division of Cardiology, Zuckerberg San Francisco General Hospital, San Francisco, CA, USA

## Abstract

Spontaneous Coronary Artery Dissection (SCAD) is an important cause of myocardial infarction that typically affects women without traditional cardiovascular risk factors. It is the most common cause of myocardial infarction in pregnant and postpartum women. SCAD is often underdiagnosed due to the lack of clinician familiarity, and patients with pregnancy-associated SCAD often have more severe clinical presentations than those without. We present a case of SCAD in a multiparous woman who presented with acute chest pain in the postpartum period.

## 1. Introduction

SCAD is a rare but important cause of myocardial infarction (MI) and sudden cardiac death. SCAD is defined as an epicardial coronary dissection not associated with atherosclerosis, trauma, or iatrogenic injury and is the most common cause of pregnancy-associated and postpartum MI [1]. SCAD typically occurs in women without traditional atherosclerotic cardiovascular risk factors, and the mechanism of injury is thought to be coronary artery obstruction from an intramural hematoma or intimal disruption [2]. Risk factors for SCAD are fibromuscular dysplasia, connective tissue disease, physical stress, emotional stress, pregnancy, and multiparity. It is hypothesized that hormonal changes during pregnancy weaken the coronary arterial vessel walls; multiparous women can accumulate these changes over several pregnancies [2].

Patients with pregnancy-associated SCAD often have more acute presentations and high-risk features than patients with non-pregnancy-associated SCAD. Most cases of pregnancy-associated SCAD occur in the first month postpartum, with the majority in the first week following delivery [3]. It is important to recognize SCAD in clinical practice as the population affected, and management is different than other causes of MI.

## 2. Case Report

A 44-year-old gravida 6 para 4 postpartum woman presented to the emergency department (ED) complaining of substernal chest pain. That afternoon, she experienced sudden-onset severe left-sided chest pain radiating to her back and shoulder. She had been discharged four days prior after induction of labor for chronic hypertension with an uncomplicated vaginal birth. Her medical history was notable only for hypertension, for which she took metoprolol. She was maintained on metoprolol with good blood pressure control for the duration of her pregnancy and after induction. The day prior to admission she was under significant emotional distress after her infant was hospitalized with hyperbilirubinemia.

**Figure 1:**
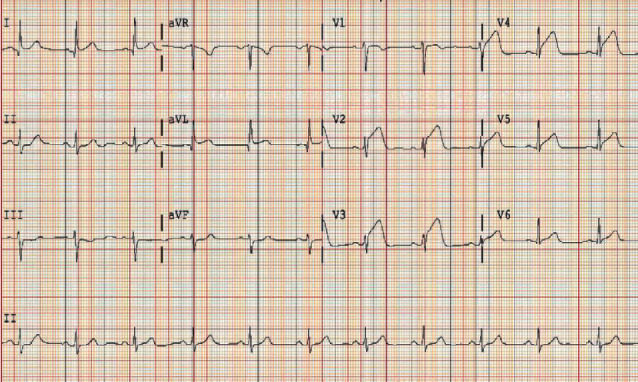
Patient's initial ECG showing ST-segment elevation in V2-V5, consistent with anterior STEMI.

In the ED, the patient was afebrile, her blood pressure was 164/101 mm Hg, her heart rate was 60 beats per minute, and she was breathing 16 breaths per minute. Her cardiovascular examination was unremarkable with normal heart sounds with no murmurs, rubs, or gallops and no jugular venous distension. Her lungs were clear, her abdomen was soft, and she had no extremity edema. Her complete blood count and comprehensive metabolic panel results were within normal limits. Her initial troponin I was 0.41 ng/mL (reference range, <0.04 ng/mL), and her electrocardiogram (ECG) (Figure 1) was significant for ST elevation in leads V2-V5 consistent with an anterior ST-segment elevation myocardial infarction (STEMI). Her chest X-ray showed normal mediastinum, no signs of cardiomegaly, and clear lungs.

**Figure 2:**
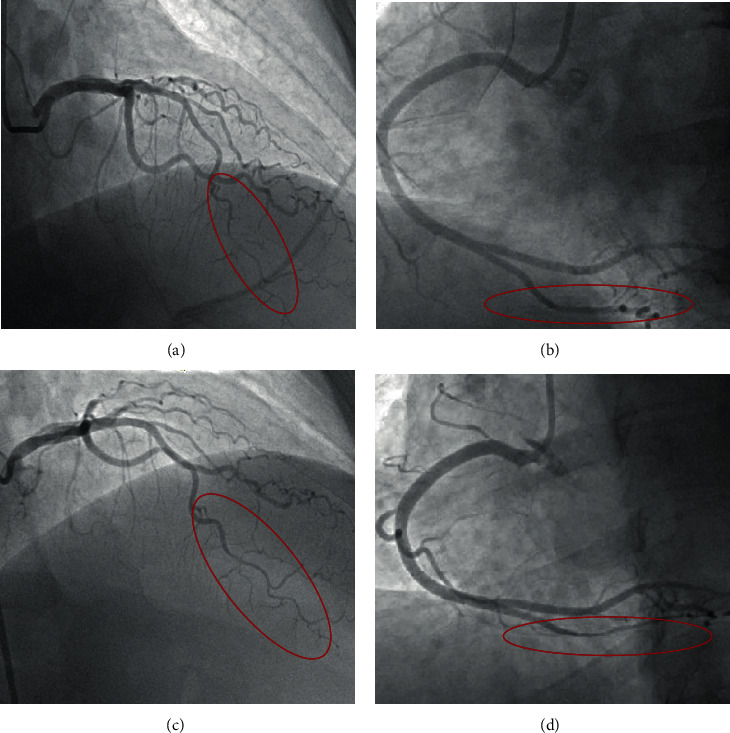
Frame A: initial angiogram showing Type 2 SCAD, with a long segment of diffuse vessel narrowing in the mid to distal LAD. Frame B: initial angiogram showing the PDA with no angiographic disease or narrowing. Frame C: repeat angiogram on hospital day 6 showing healing of initial SCAD lesion after medical therapy. Frame D: repeat angiogram showing new Type 2 SCAD lesion in the mid-PDA.

Given the concern for acute coronary syndrome (ACS), the patient was emergently taken to the cardiac catheterization lab. Her coronary angiogram showed haziness of the midleft anterior descending (LAD) artery followed by an abrupt change in arterial caliber extending to the distal LAD and diagonal branches (Figure 2(a)). There was no clear visualization of a dissection flap, but there was a visible long segment of diffuse vessel narrowing in the mid to distal LAD highly suspicious for coronary dissection. Contrast flow in the distal vessel was normal (TIMI 3), and there were no luminal changes with the administration of intracoronary nitroglycerin. There was no angiographic evidence of atherosclerotic disease in any of her coronary arteries, and no evidence of narrowing in the coronary arteries outside of the LAD. Her chest pain was resolved during the procedure, and her ECG showed normalization of her ST elevation. Given the patient's hemodynamic stability, normal blood flow in the distal LAD, resolution of chest pain, and suspicion for dissection, she was managed conservatively.

**Figure 3:**
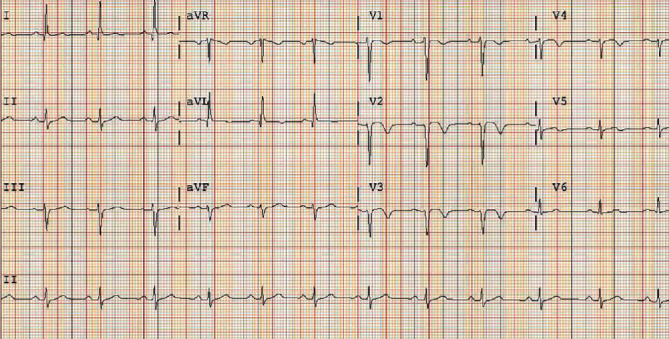
Repeat ECG on day 2 of hospitalization showing the expected evolution of anterior STEMI with QS waves and T-wave inversions seen in V2-V5.

She was started on IV nitroglycerin for hypertension, metoprolol, and dual antiplatelet therapy with aspirin and clopidogrel and then transferred to the coronary care unit for close observation. Her transthoracic echocardiogram showed normal left ventricular function with an ejection fraction of 68% and hypokinetic septal and apical walls in the distribution of the LAD. Her repeat ECG (Figure 3) showed the expected evolution of her STEMI with QS waves and T-wave inversions seen in leads V2-V5. She was weaned off the nitroglycerin and transitioned to captopril and nifedipine for strict blood pressure control and safety with breastfeeding.

She remained chest-pain free during the course of the hospitalization and underwent a repeat angiogram to evaluate for the progression of disease given the diagnostic uncertainty. Her repeat angiogram on day 6 of hospitalization showed complete resolution of the LAD lesion (Figure 2(c)) consistent with dissection. Compared to the initial angiogram, the area of haziness was no longer present, and the vessel tapered appropriately towards the apical LAD. However, a new tubular narrowing with abrupt vessel caliber change (Figure 2(d)) was noted in the midposterior descending artery (PDA), compared to her initial angiogram (Figure 2(b)) with normal flow and no clear dissection flap. This event was concerning for a new dissection event. The patient was asymptomatic throughout her hospitalization and was discharged home with close obstetric, primary care, and cardiology outpatient follow-up.

## 3. Discussion

In this postpartum woman presenting with a STEMI with angiographic findings suggestive of coronary dissection and no evidence of atherosclerotic disease, a diagnosis of SCAD was made. SCAD commonly presents as ACS with ST-segment changes, and early angiography should be performed to confirm the diagnosis, assess the coronary anatomy, and facilitate revascularization when necessary [2–4]. SCAD is often misdiagnosed and a research group recently published a flowchart to aid in the diagnosis [5]. Angiographically, SCAD has three main classifications: Type 1 is identified by contrast staining showing multiple lumens, Type 2 is an abrupt change in vessel caliber with diffuse tubular stenoses of varying length, and Type 3 is a focal stenosis that mimics atherosclerosis and intracoronary imaging using intravascular ultrasound or optical coherence tomography is often required to confirm the diagnosis. The angiographic appearance of Type 2 is often missed and shorter lengths, such as 20-30 mm, may require repeat angiography or intracoronary imaging to make the diagnosis [4, 6]. The patient in this case had a Type 2 SCAD lesion with suggestive features on the initial angiogram that was confirmed with a repeat angiogram showing healing of her lesion. The LAD is the most common artery affected in both pregnancy-associated and non-pregnancy-associated SCAD [2, 3]. Patients with pregnancy-associated SCAD often have more severe clinical presentations than non-pregnancy-related SCAD with multivessel dissections and acute heart failure [3].

Once a diagnosis is made, SCAD consensus guidelines recommend a conservative approach in patients who are hemodynamically stable and without active ischemia [2, 4, 6]. Invasive management in SCAD is associated with a high incidence of iatrogenic coronary dissection. Patients should be initiated on aspirin, beta-blockade, and additional antiplatelet therapy with clopidogrel for up to 12 months after the initial event. The role of antiplatelet therapy in SCAD is unclear but is extrapolated from its evidence in acute coronary syndrome and may be beneficial with prothrombotic intimal tears. Beta-blockade is thought to reduce arterial shear stress and may be beneficial through mechanisms similar to that of the treatment of aortic dissection [6]. The majority of SCAD patients (70-90%) managed conservatively have spontaneous healing of their dissection. However, patients with hemodynamic instability, electrical instability, ongoing/recurrent ischemia, abnormal blood flow (TIMI 0-1), and/or left-main coronary artery dissection should be considered for urgent revascularization [2].

Due to the risk of early dissection recurrence or extension, SCAD patients should be observed inpatient for 2-4 days until asymptomatic and clinically stable [4, 6]. Patients with pregnancy-associated SCAD have been found to have a higher prevalence of multivessel dissection events [3]. The patient had a new dissection event on her repeat coronary angiogram but remained chest-pain free without any new evidence of ischemia. There is growing evidence into the role that pregnancy and future pregnancy play with SCAD recurrence and the rates of recurrence have been reported to be as high as 30 percent [7]. This creates a relevant ethical question, and a recent experience-based survey reported that the majority of responders discourage future pregnancy after SCAD [8]. Patients who decide to proceed with pregnancy should have dedicated care with cardiologists and obstetric specialists [7].

## 4. Conclusion

SCAD is an important and underdiagnosed cause of MI associated with pregnancy. Clinicians should maintain high suspicion in women presenting with chest pain in the postpartum period. The diagnosis of SCAD should be made with early angiography, and a conservative approach with medical management is recommended if possible. All patients with SCAD should be followed closely outpatient as researchers learn more information on the factors associated with recurrence.
